# Human-Mediated Dispersal of Seeds by the Airflow of Vehicles

**DOI:** 10.1371/journal.pone.0052733

**Published:** 2013-01-08

**Authors:** Moritz von der Lippe, James M. Bullock, Ingo Kowarik, Tatjana Knopp, Matthias Wichmann

**Affiliations:** 1 Centre for Ecology and Hydrology, Wallingford, United Kingdom; 2 Department of Ecology, Technische Universität Berlin, Berlin, Germany; 3 Department of Animal Ecology, Universität Potsdam, Potsdam, Germany; 4 Biodiversity and Systematic Botany, Universität Potsdam, Potsdam, Germany; University of Tartu, Estonia

## Abstract

Human-mediated dispersal is known as an important driver of long-distance dispersal for plants but underlying mechanisms have rarely been assessed. Road corridors function as routes of secondary dispersal for many plant species but the extent to which vehicles support this process remains unclear. In this paper we quantify dispersal distances and seed deposition of plant species moved over the ground by the slipstream of passing cars. We exposed marked seeds of four species on a section of road and drove a car along the road at a speed of 48 km/h. By tracking seeds we quantified movement parallel as well as lateral to the road, resulting dispersal kernels, and the effect of repeated vehicle passes. Median distances travelled by seeds along the road were about eight meters for species with wind dispersal morphologies and one meter for species without such adaptations. Airflow created by the car lifted seeds and resulted in longitudinal dispersal. Single seeds reached our maximum measuring distance of 45 m and for some species exceeded distances under primary dispersal. Mathematical models were fit to dispersal kernels. The incremental effect of passing vehicles on longitudinal dispersal decreased with increasing number of passes as seeds accumulated at road verges. We conclude that dispersal by vehicle airflow facilitates seed movement along roads and accumulation of seeds in roadside habitats. Dispersal by vehicle airflow can aid the spread of plant species and thus has wide implications for roadside ecology, invasion biology and nature conservation.

## Introduction

Human-mediated dispersal (HMD) is a driver of long-range spread of plant species and is increasingly gaining attention in dispersal [1] and invasion ecology [2], [3]. Roadsides are particularly relevant in terms of HMD as some invasive species expand their ranges rapidly along road networks [4], [5]. At the landscape scale, several studies have demonstrated that the density of human transportation corridors [6], [7] or human use of roads [8] are related to the frequency or spread rate of non-native plants.

HMD by vehicles has only been studied in a few contexts to date. Several studies have demonstrated potential dispersal through attachment to vehicles by finding seeds of many species in samples of mud from the surface of vehicles [9]–[13]. Only recently, the spatial reach of dispersal by seeds attaching to motor vehicles has been quantified, showing that long-distance dispersal over more than 256 km is achieved by a sizeable proportion of seeds that become attached with mud on cars [14]. Second, studies of seed deposition along roadsides have considered the combined roles of attachment and airflow by vehicles for dispersal. To exclude non-traffic-related dispersal, seeds have been collected in long motorway tunnels [15], [16] and seeds of known cultivars of oilseed rape/canola have been exposed on the roads and their offspring traced with molecular markers [17]. Gaps remain, however, in our understanding and in the quantifications of the mechanisms of vehicle-related seed transport. For example, to our knowledge, there has been no quantification of the dispersal kernel of seeds transported by cars (but see [14]), including the distribution of dispersal distances. In addition, the role of slipstreams and air turbulence caused by passing vehicles [18] has been suggested to be important in the spread of roadside plant populations [17] but this process has not yet been studied.

Turbulent airflow is reported by studies of the aerodynamics of vehicles [18]. A narrow zone around a passing vehicle is affected by turbulence, caused by flow separation from the boundary layer at the surface of the moving vehicle. Although it has a stochastic nature, the mean direction of this turbulence leads to a characteristic flow field around the moving vehicle. Besides turbulent airflow close to the vehicle, it is primarily the slipstream in the wake of the passing vehicle that affects airflow in the direction of the traffic movement. This slipstream acts over a distance which is determined as a multiple of the vehicle height [19], [20]. The airflow in this “far wake region” is caused by a velocity deficit in the wake of the moving vehicle and tends to be laminar. Previous studies of seed dispersal by wind [21] suggest that vehicle's airflow could influence the dispersal of seeds along roads.

A better mechanistic understanding through experimental quantification has been achieved for some seed dispersal processes including wind dispersal [21]–[26] and, to some extent, animal dispersal [27]–[31]. While wind and also animals often act as vectors for primary dispersal, humans, as in our experiment, normally trigger secondary dispersal. For HMD, kernels have recently been quantified experimentally for dispersal on footwear [1], [2] and cars [14]. The results indicate that HMD may follow well defined mechanisms and that dispersal distances can far exceed primary dispersal vectors. To expand on earlier results on dispersal by human activity, we aim in this paper to extend experimental quantification of HMD to that by airflow of vehicles.

By quantifying dispersal kernels for four species, we determine the effect of repeated vehicle passes on the parallel and lateral movements of seeds. We hypothesize that: i) repeated vehicle passes result in continued secondary dispersal of seeds along the road; ii) turbulent airflow around passing vehicles causes a lateral movement of seeds towards the road verge; and iii) the extent of seed transport by passing vehicles depends on seed traits, such as morphology. To test these hypotheses we set up an experiment in which a car was repeatedly driven through lines of marked seeds comprising four species. Distance and direction of seed movement was quantified.

## Methods

### Ethics Statement

The study took place on a private parking ground and was permitted by Centre of Ecology and Hydrology at Winfrith Technology Centre, Winfrith, Dorset, UK. No specific permits and approvals were required, as the speed of car driving during the experiment did not exceed the maximum speed allowed in the area. No seeds of protected species were used. Three of our study species commonly occur in the study area but to avoid any risk of establishment of the fourth species, we applied a microwave treatment to the seeds of *Ambrosia artemisiifolia* to prevent any seeds lost from the experimental site from germinating.

### Experimental Design

Our experimental design focussed on seed dispersal by the slipstream of a car. Therefore experiments were performed during dry and still weather conditions to exclude dispersal by water-aided adhesion or by ambient wind alone. To quantify the effect of multiple dispersal events caused by a sequence of passing vehicles, a medium sized estate car (Vauxhall Astra) was driven repeatedly along a road section where marked seeds were laid out.

We carried out this study on a tarmac surface parking area (coordinates: 50.68° north, 2.26° west) 120 m in length and 20 m in width, with a concrete kerb 7 cm in height, and bordered by a gravelled verge. The length of the parking area was divided into a 60 m acceleration zone, a 45 m distance over which seed dispersal was measured, and a final 15 m for deceleration and turning. The acceleration zone was used to achieve a speed of 48 km/h (30 mph) which was kept constant through the seed dispersal zone. This equals the maximum speed allowed in urban areas throughout many European countries.

**Figure 1:**
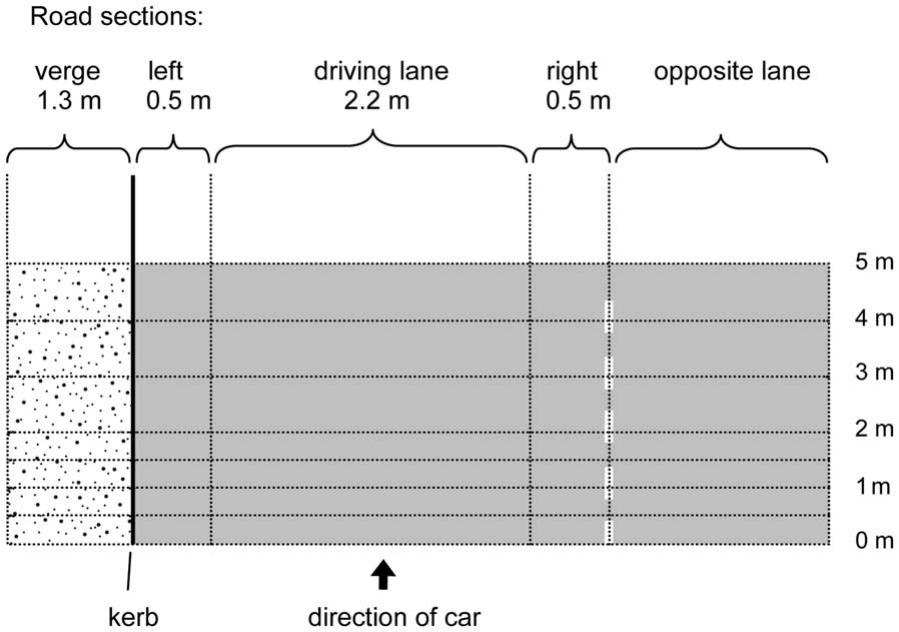
Sections of the study road. The car remained within the driving lane, making this the section most directly impacted by the passing vehicle. The adjoining sections (left and right) were not driven over, but would have been strongly affected by the vehicle's airflow. Seed settlement is considered to occur in the verge, which provides the most immediate establishment opportunity for seeds. The opposite lane represents the area beyond the midpoint of the lane, where, on a real road, the seed would be in the path of oncoming traffic.

Longitudinal dispersal was measured using intervals marked perpendicularly across the traffic lane (Fig. 1). The first 2 m were marked at 0.5 m intervals, metres 2–10, as well as one meter before starting line were marked at 1 m intervals, and metres 10–45 were marked at 5 m intervals. We also divided the lane into five sections parallel to the direction of car movement to track lateral movement of seeds (Fig. 1).

### Study species and tracking seeds

**Figure 2:**
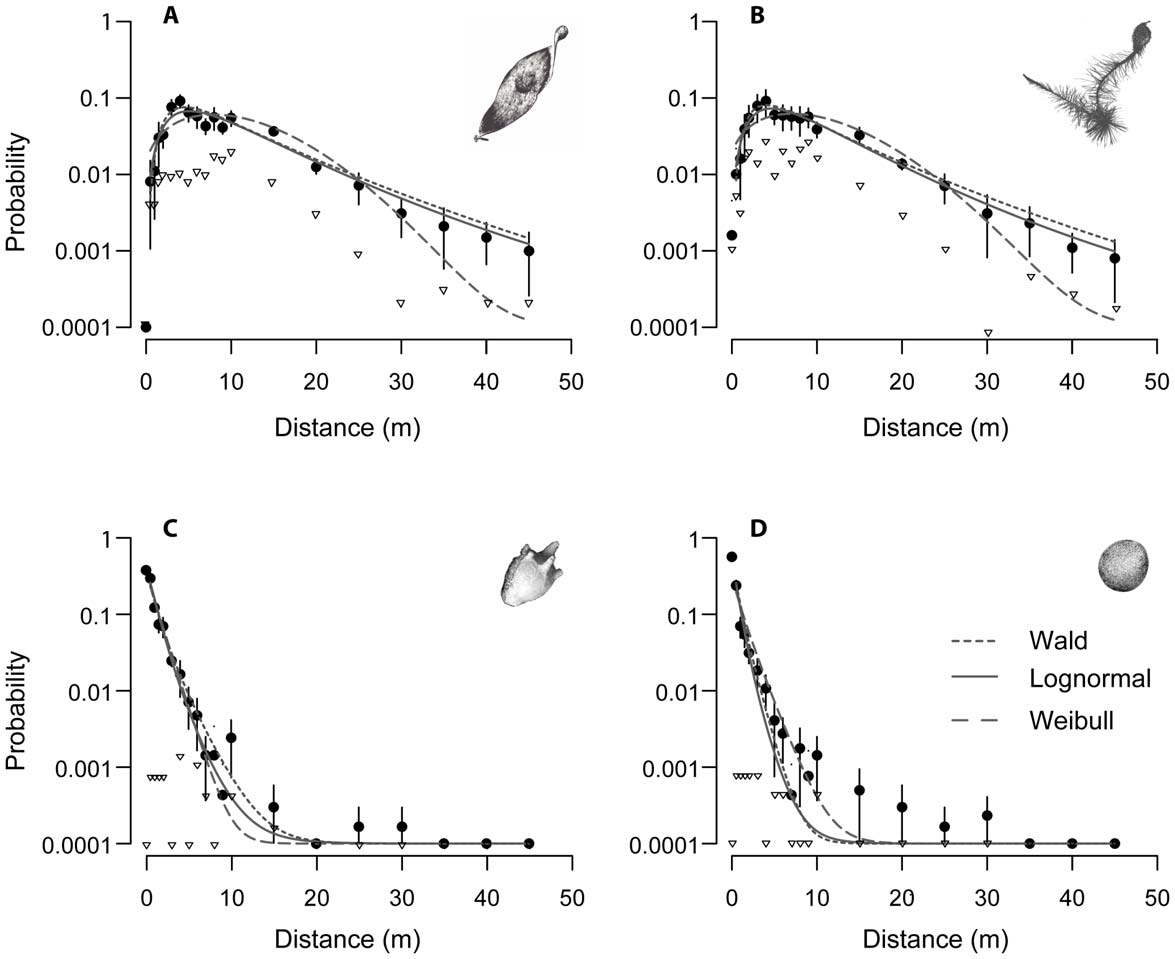
Probability distributions of seeds along the study road after 80 vehicle passes. (A) *Ailanthus altissima* (B) *Clematis vitalba*, (C) *Ambrosia artemisiifolia* and (D) *Brassica napus*. Filled circles represent the mean (±95% CI) probabilities of 10 replicates. The grey lines show the fitted dispersal functions. Open triangles show mean probabilities for seeds to reach the road verge at that distance along the line of travel. The y-axis is on a log scale and 0.0001 was added to all probabilities to show zero values. Note that this is not a total dispersal kernel but solely shows secondary dispersal by car's airflow.

We used four species known to be common along roads or whose spread has been linked to vehicle movement (Table 1): *Ailanthus altissima* (Mill.) Swingle, *Ambrosia artemisiifolia* L., *Brassica napus* L., *Clematis vitalba* L.. In the following we will refer to these species by their genus name. These species are invasive at least in parts of Europe and are assumed to affect biodiversity and/or human health. The species were also selected to represent different seed morphologies (with and without appendices for wind dispersal, Fig. 2) to allow the influence of seed traits on dispersal by vehicles to be assessed.

**Table 1:** Characteristics of the study species.

Species	Seed morphology	Falling velocity (m/s)	Association to transport corridors
*Ailanthus altissima*	Winged	0.84 (0.75)	Rapid range expansion along transport corridors in the introduced range and negative impacts on adjacent habitats [50]
*Clematis vitalba*	Plumed	0.91 (0.85)	Rapid range expansion along transport corridors in native and introduced range, negative impacts on native forests [51]
*Brassica napus*	Smooth	3.8 (3.8)	Spread along roads, risk of escape of genetically modified lineages [46], [48]
*Ambrosia artemisiifolia*	Hooked	3.7 (3.7)	Severe allergenicity to humans, rapidly spreading along roads in Europe [45]

Seed morphology, mean falling velocity of painted seeds (10 replicates, in brackets: unpainted seeds) and association to transport corridors of the four study species.

All seeds were coloured with a fluorescent paint to allow them to be tracked during sequential dispersal events. To minimise weight gain of the seeds, we used fluorescent airbrush colours, applied with a spraying gun of 0.3 mm needle size (see [32] for details). To test the influence of colour application on wind-dispersal ability, the falling velocities of fifteen seeds of each species before and after colour application were measured using the equipment and method described in Askew et al. [33]. Paint application had a significant but small effect on falling velocity (Table 1). Falling velocity increased on average by 0.9% in *Brassica* and 1.3% in *Ambrosia*. Higher increases were observed for *Clematis* (7.1%) and *Ailanthus* (12.0%). This increase in falling velocities due to seed weight gain probably reduced dispersal distances, making our estimates somewhat conservative.

### Dispersal experiments

In a first experiment, we placed 300 seeds each of *Ambrosia* and *Brassica* and 200 seeds each of *Ailanthus* and *Clematis* at the start line (0 m) of the 45 m dispersal zone. Seeds were spread evenly across the driving lane over a narrow strip extending 30 cm in front of the starting line.

The car was then driven repeatedly along the driving lane in a sequence of up to 80 “passes” in one direction. The car left the parking area to return to the starting point to avoid disturbing the seeds. Five sequences with different numbers of passes were tested—1, 10, 20, 40 or 80—with the lower numbers serving as the basis for the next sequence (i.e., to reach 10 passes, we recorded the seed distribution after 1 pass and then did another 9 passes). Each set of sequences was replicated 10 times on different days.

At the end of each sequence (1; 10; 20; 40; 80), seed distribution over the grid formed by the perpendicular and parallel sections (see Fig. 1) was recorded. We searched for and located seeds using a strong LED-UV lamp [32] and so the experiment was performed at dusk, night and dawn. If any seed movement by the ambient wind was observed during seed searching, the trial was terminated and the data for this entire replicate discarded. We visually checked the car but never found any attached seeds. To get a better understanding of the trajectories of seeds in the airflow of the car, we filmed the first pass of the vehicle several times with a digital video camera at 30 frames/s.

In a second experiment we focussed on the lateral movement of the seeds using only two species of different seed morphology. Seeds of *Ailanthus* and *Brassica* were coloured with five different colours; each colour group of seeds was laid out in a different parallel road section (Fig. 1). At the 0 m line we placed 50 seeds in the driving lane and 25 in each of the other sections for *Ailanthus*, and 100 and 50 seeds respectively for *Brassica*. After a single pass, the positions of all seeds in the parallel and perpendicular sections were recorded and the seeds were collected. Any possibly remaining seeds were removed by cleaning the road with a professional vacuum cleaner. This procedure was replicated 50 times for each species.

### Data analysis

To standardise the frequencies across the differently sized perpendicular sections, we divided the frequencies of the 5 m sections by 5 and multiplied those of the 0.5 m sections by 2; i.e. all frequencies were standardised in terms of the 1 m sections. We fitted empirical dispersal models to the probability distributions of seeds along the road transect. Distance was represented as the longitudinal distance between the starting line and the end of each section. We chose three functions that are frequently used to model wind dispersal of seeds [34], [35]: the Weibull, the Lognormal and the inverse Gaussian (Wald) functions. These functions were fitted to the probability data by generalised non-linear regression using the Proc NLMIXED function in SAS 9.2. This uses a maximum likelihood fitting procedure and is able to deal with non-normal errors; in this case a binomial distribution was used. The equations used were those supplied by Jongejans et al. [35]:

1. Wald: 
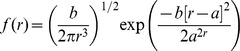

2. Lognormal: 
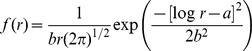

3. Weibull: 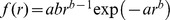

Where *f* (*r*) is the probability that a seed is dispersed to a specific distance *r*, *a* is the scale parameter and *b* the shape parameter of the probability density functions (note that *a* and *b* have different values in each equation). We used Akaike's Information Criterion (AIC) to compare the fits of the different functions.

For the experiment on the lateral movement of seeds, we calculated the probability of reaching a certain section of the study road separately for the seed groups of each starting section. Probabilities were averaged over the 50 replicates and were displayed in a grid plot using the function ‘image’ (package ‘graphics’) of the statistical and programming environment R 2.10 [36].

Videos of the trajectories of seeds in the wake of the car were analysed by slow motion and single frame playback. We visually estimated the height of seed lift above the ground by relating it to the height of different parts of the vehicle.

## Results

### Dispersal distances and kernels

Mean recapture rates (±1SD) of viable diaspores after one vehicle pass were 94.5% (±4.9) for *Ailanthus*, 93.2% (±3.2) for *Clematis*, 93.8 (±1.4) for *Brassica* and 90.6% (±4.4) for *Ambrosia*. Losses were mainly due to seeds being crushed by tyres. Recapture rates decreased with increasing numbers of vehicle passes to 84.5% (±6.7) for *Ailanthus*, 84.6% (±11.4) for *Clematis*, 80.7% (±6.8) for *Brassica* and 72.0% (±5.8) for *Ambrosia* after 80 rounds.

**Figure 3:**
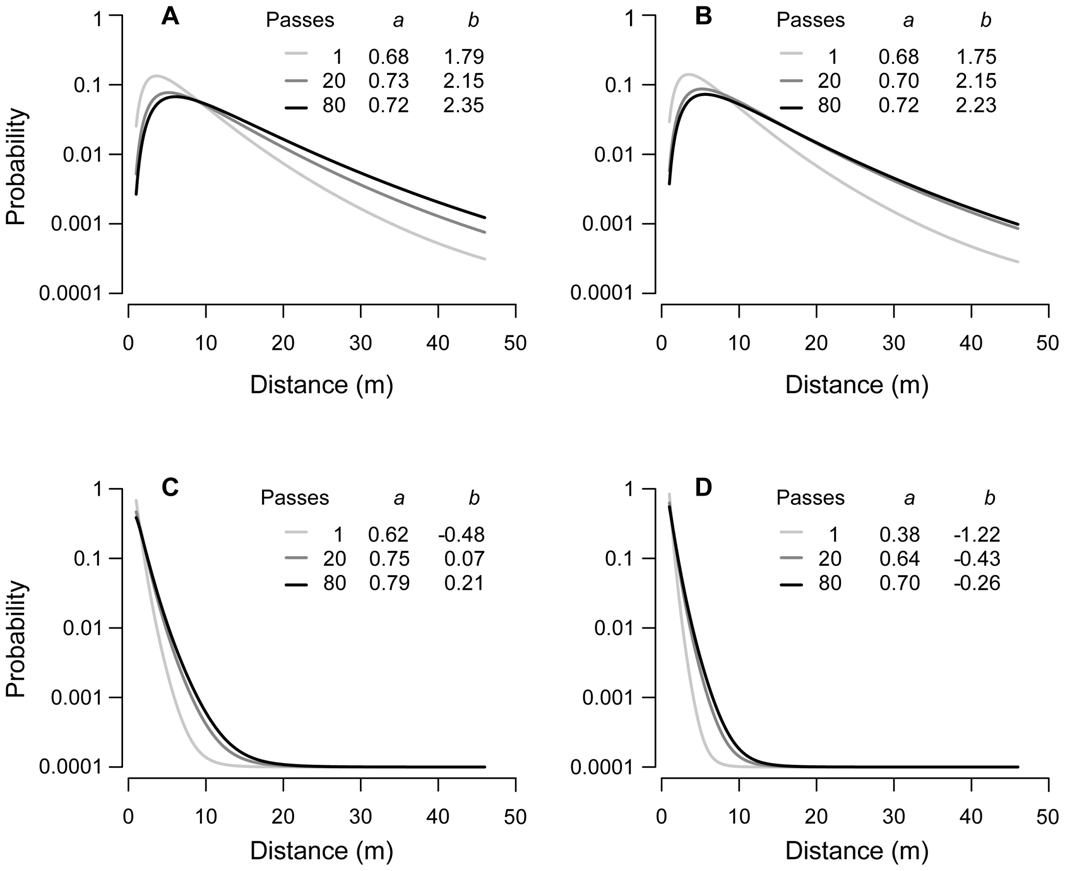
Effect of the number of vehicle passes on the shape of the fitted dispersal kernel (Lognormal function). (A) *Ailanthus altissima*, (B) *Clematis vitalba*, (C) *Ambrosia artemisiifolia*, and (D) *Brassica napus*. The y-axis is on a log scale and 0.0001 was added to all probabilities to show zero values. Inset tables show parameter estimates (*a* = scale parameter, *b* = shape parameter) for the different numbers of vehicle passes.

As the number of car passes increased, mean, median and 99% percentile of the dispersal distance of recaptured seeds increased but the relative effect diminished (Table 2). Probability distributions and mean dispersal distances were markedly similar for *Ailanthus* and *Clematis*, and for *Brassica* and *Ambrosia* (Figs. 2, 3; Table 2).The probability distributions of both *Ailanthus* and *Clematis* after 80 vehicle passes describe humped curves with the modes at approximately 5 m and a long tail extending over the entire 45 m section (Fig. 2a, b). As a measure of long distance dispersal, the 99% percentile of the probability distributions increased strongly from the first to ten passes in these species and remained constant at 40 m for 20 passes and more. In contrast, probability distributions for *Brassica* and *Ambrosia* after 80 passes peaked at the starting line (0 m) and then declined sharply up to a maximum distance of 30 m (Fig. 2c, d). We observed that both of these species were predominantly dispersed in the two narrow strips where the tyres passed. The 99% percentiles of both species reached 10 m (*Ambrosia*) and 15 m (*Brassica*) after 80 passes.

**Table 2:** Dispersal distances for the study species after different numbers of vehicle passes.

# vehicle passes	1	10	20	40	80
***Ailanthus altissima***					
Mean	5.14±3.07	9.85±7.97	9.95±7.84	10.33±7.96	10.83±8.11
Median	5	8	8	8	8
99^th^ percentile	15	40	40	40	40
***Clematis vitalba***					
Mean	4.95±3.43	9.19±7.52	9.94±7.93	10.08±8.03	10.46±8.00
Median	4	7	8	8	8
99^th^ percentile	15	35	40	40	40
***Brassica napus***					
Mean	0.98±1.12	1.26±1.88	1.60±2.62	1.59±2.66	1.71±2.87
Median	0.5	0.5	0.5	0.5	1
99^th^ percentile	4	8	12	10	15
***Ambrosia artemisiifolia***					
Mean	1.04±1.07	1.38±1.85	1.51±1.92	1.64±2.03	1.59±2.05
Median	0.5	0.5	1	1	1
99^th^ percentile	5	9	9	9	10

Note our recapture rates given under ‘results’. Also note that movement along the road decreased considerably for multiple vehicle passes but resulted in increased lateral transport. Dispersal distances might change greatly when dispersal by car's airflow interacts with other forms of dispersal (e.g. wind) transporting seeds back to the driving lane.

At the road verge, the seed densities for *Ailanthus* and *Clematis* after 80 passes followed a similarly shaped distribution as for all parallel sections combined, but were about an order of magnitude lower (Fig. 2). For *Brassica* and *Ambrosia*, dispersal to the road verge occurred only occasionally, and within the first 10 m.

In the video analysis (see supporting information, Video S1), only trajectories of seeds of *Ailanthus* and *Clematis* were clearly visible. While the car was passing, a small proportion of the seeds were blown to both sides of the road. As soon as the car had completely passed the line of seeds, a strong linear movement of seeds in the driving direction was seen. Most seeds that were picked up by the slipstream were briefly lifted approximately 50 cm and then fell back to the ground, where they tumbled after the car.

Probability distributions were best fit by the Wald and the Lognormal function. This result was similar across all four species despite the very different seed distributions along the road. However, differences in AIC were generally low between all the dispersal functions tested. For *Ailanthus* and *Clematis* the Wald function resulted in a marginally lower AIC, whereas the Lognormal function better described the dispersal tails (Fig. 2). The Wald function slightly overestimated the tails of the kernels while the Weibull clearly underestimated them, despite having a relatively low AIC. Estimated shape parameters for all functions were very similar for *Ailanthus* and *Clematis* (Table 3). For *Ambrosia* and *Brassica*, Lognormal functions fitted dispersal kernels only at a slightly smaller AIC than the Wald function. All functions predict very low probabilities for both species to disperse more than 20 m. (For more details see supporting information, Table S1)

**Table 3:** Parameter estimates and AIC for different dispersal functions.

Species	Model	*a*	*b*	AIC
***Ailanthus altissima***				
	Weibull	1.858	0.008	44.0
	Lognormal	0.724	2.307	43.2
	**Wald**	20.456	12.952	43.1
***Clematis vitalba***				
	Weibull	1.779	0.011	46.3
	Lognormal	0.724	2.229	45.5
	**Wald**	18.366	12.275	45.4
***Brassica napus***				
	Weibull	0.852	0.975	40.7
	**Lognormal**	0.697	−0.260	34.7
	Wald	1.621	1.089	35.2
***Ambrosia artemisiifolia***				
	Weibull	0.976	0.847	39.9
	**Lognormal**	0.791	0.211	39.6
	Wald	1.270	1.357	39.7

Models were fit to the probability distributions for the four study species after 80 vehicle passes. The model with the lowest AIC for each species is in bold.

The shape of the dispersal kernels changed with increasing vehicle passes. At higher numbers of passes, the fitted kernels for *Ailanthus* and *Clematis* show similar modes, but a fatter tail (Fig. 3). For *Ambrosia* and *Brassica* the decline in the dispersal kernels was less steep at higher numbers of passes and reached a probability of zero at a distance approximately 5 m further from the starting point compared to the first vehicle pass. The change in the shape of the dispersal kernel for all species was most pronounced between 1 and 20 rounds while the changes between 20 and 80 rounds were less pronounced.

### Lateral movement of seeds

**Figure 4:**
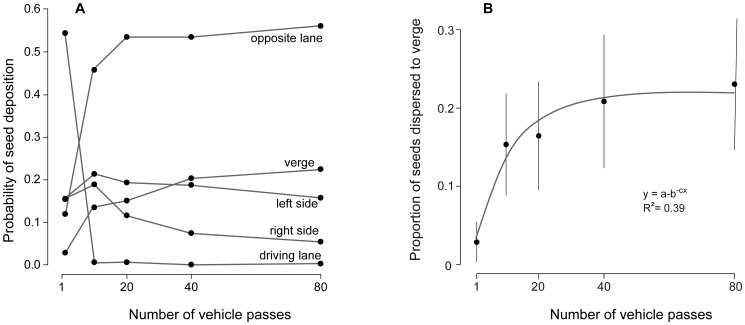
Lateral movement of seeds of *Ailanthus altissima* in relation to the number of vehicle passes. (A) Mean probabilities of seed deposition of *Ailanthus altissima* in the parallel road sections in relation to the number of vehicle passes. (B) Proportion (±95% CI) of seeds of *Ailanthus altissima* accumulating at the verge in relation to the number of vehicle passes. At the start of experiment seeds were laid out only on the driving lane.

In the first experiment, we observed considerable lateral transport of seeds to the verge in *Ailanthus* and *Clematis*, but not in the other species (Fig. 2 a, b). There was a rapid accumulation of seeds at both sides of the driving lane (Fig. 4). After 10 vehicle passes, only a small proportion (<4%) of *Ailanthus* seeds remained in the driving lane (Fig. 4a; similar results were found for *Clematis* and are not shown). Seeds accumulated rapidly in the opposite lane, in the left lane along the kerb, and in the road verge, but the right lane showed negligible accumulation. Accumulation of seeds in the road verge increased asymptotically with the number of vehicle passes, reaching a plateau of about 20% of seeds after 40 passes (Fig. 4b).

**Figure 5:**
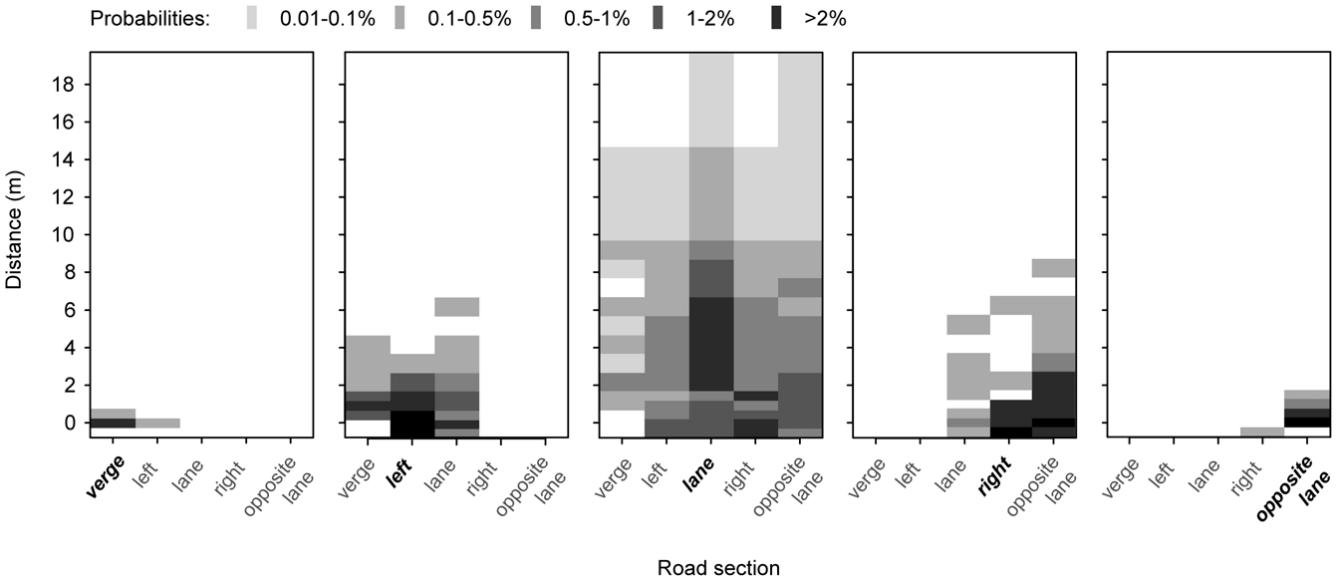
Influence of the starting section on parallel and lateral movement of seeds by a passing car. Dispersal patterns of *Ailanthus altissima* seeds after one vehicle pass for seeds initially placed in the verge (left panel), the left side (second panel from left) the driving lane (centre panel), the right side (second panel from right) and the opposite lane (right panel). Probabilities of reaching the different sections of the study road are indicated by different grey shading. Probabilities are averaged over 50 replicates.

The second experiment clarified the lateral transport of seeds of *Ailanthus* that were laid out at different starting sections of the study road (Fig. 5). Seeds placed at the road verge moved only slightly, whereas the seeds placed in the driving lane were transported farthest across the road and reached all other parallel sections with high probabilities. Seeds put into the left or right section adjoining the driving lane travelled only to the immediately neighbouring sections and were transported over much shorter distances.

In contrast to *Ailanthus* and *Clematis*, seeds of *Brassica* and *Ambrosia* largely remained in the driving lane (Fig. 2). Even after 80 rounds of vehicle passes, fewer than 10% of seeds of either species were transported to the side lanes and only a negligible proportion (<1%) reached the road verge or the opposite lane (Fig. 2c, d). The second experiment confirmed very low lateral transport for seeds of *Brassica*.

## Discussion

Previous research has revealed that vehicles serve as agents of long-distance dispersal either in terms of long-lasting attachment of seeds [10], [13], [15] or through spillage during transport of goods [37], [38]. Our results add the mechanism of repeated stepwise dispersal of seeds over road surfaces by the airflow of passing vehicles. Dispersal by airflow may interact with other dispersal vectors, traffic related as well as wind [39] and animals, which has considerable implications for long distance dispersal. The high rates of recaptured seeds on the ground demonstrate that under dry weather conditions attachment of seeds to vehicles is low. Our video analysis revealed that the majority of seeds from species with morphological adaptations to wind dispersal were indeed transported with the slipstream of the passing car.

### Dispersal distances

The distances we observed for secondary dispersal by vehicle airflow are on a scale similar to the primary dispersal distances reported for our study species. Soons and Ozinga [40] modelled a median distance of 10.1 m and a 99^th^ percentile of 100 m for wind dispersal of *Clematis*. Matlack [41] observed a maximum distance of 112 m for wind dispersal of *Ailanthus*. Whereas the median distance is comparable, these values are approximately double those for secondary dispersal by a vehicle after 80 passes. Colbach et al. [42] found a mean primary dispersal distance of 0.5 m and a 99^th^ percentile of 1.6 m for *Brassica*, which amounts to approximately half to one tenth the respective values for secondary dispersal observed in this study. Our results give experimental evidence that distances of seed dispersal by car's airflow can be equal and even higher compared to natural dispersal.

Dispersal by means of seeds attaching to the surface of vehicles is affected by seed size and seed mass [10], [13]. In this study, falling velocity triggered by morphological adaptations for wind dispersal appeared to be important. Species with plumes (*Clematis*) or wings (*Ailanthus*) were moved over much greater mean distances than species without such adaptations (Table 1). Figure 2 demonstrates strikingly congruent dispersal kernels for the species with wind dispersal morphologies, and also for the two species without such morphologies. This coincides with similar falling velocities of *Ailanthus* and *Clematis*, and of *Brassica* and *Ambrosia*, respectively suggesting that falling velocity influences secondary dispersal by vehicle airflow as it does for primary wind dispersal [33].

For *Brassica* and *Ambrosia* our observation of a higher degree of seed movement in the areas traversed by tyres might suggest short contact with tyres rather than airflow accounts for dispersal. Although we cannot separate the effect of tyre contact and airflow in this experiment, the different shapes of the dispersal kernels for *Ailanthus* and *Clematis* versus *Brassica* and *Ambrosia* suggest different processes with the former two species being more influenced by the airflow.

### Airflow dispersal of species with morphological adaptation to wind dispersal

For *Ailanthus* and *Clematis* the shape of the dispersal kernel and the strong lateral movement of seeds even from sections adjoining the driving lane indicate a dominant effect of vehicle airflow on the dispersal process. Solazzo et al. [20] found a sizeable effect of vehicle airflow on the distribution of particulate matter along roads, primarily caused by the slipstream of passing cars. Likewise, the slipstream of the car appeared to move the seeds in our experiment almost exclusively in the direction of traffic. As a second process, sideward eddies near the ground [18] and sideways components of the airflow [19] are likely to cause a simultaneous strong lateral dispersal to each side of the driving lane. This lateral movement takes seeds away from the influence of subsequent vehicles. Although this process greatly decreases longitudinal dispersal distances, it effectively transports seeds from the road surface to potentially suitable establishment sites at the road verge. This suggests a strong effect of vehicle airflow on realised dispersal, i.e. the proportion of dispersed seeds that can germinate and establish successfully. However, secondary dispersal by ambient wind [39] may move some seeds back onto the road where they are available for further dispersal by airflow, again.

The mechanisms behind the strong lateral movement are partially revealed by our additional experiment for *Ailanthus*. Seeds placed in the driving lane showed a wide lateral dispersion after the vehicle passed, while seeds placed in the adjoining sections showed shorter transport distances and lower overall probabilities of lateral transport (Fig. 4, 5). Repeated passes by vehicles will therefore result in a higher lateral transport from the driving lane to adjoining sections rather than towards the driving lane. This is in accordance with the low lateral reach of the slipstream effect in the wake of a moving vehicle in contrast with the significant airflow behind, which extends over a distance of 40 times the vehicle height [19]. While most commonly seeds might be starting at the road verge they are least likely to travel long distance by car's airflow. Conversely the highest probabilities of long distance dispersal have those seeds exposed on the driving lane. Yet, the number of these seeds strongly depends on primary and secondary dispersal by other vectors.

A mechanistic model for wind dispersal over the ground has been introduced and successfully validated with experimental data [25]. This model describes dispersal distances over a given time span as a function of wind drag and friction of the surface that a seed is experiencing. The airflow caused by passing vehicles has also been successfully modelled by computational fluid dynamics [20]. Thus, if the horizontal wind vector of a vehicle in motion and the friction of the road surface are analysed, a fully mechanistic description of vehicle dispersal might be obtained. However, as indicated by the video analysis (see supporting information, Video S1), tumble dispersal over the ground is only part of the process for wind dispersed species. Another is in-air wind dispersal once the seeds have been lifted by the turbulence caused by the passing car. Turbulence and updrafts caused by ambient wind have been shown to be important in long-distance primary dispersal by wind [22], [24], [43], [44]. Updrafts are probably caused by the turbulences close to the passing car where several upward and sideward eddies occur [18]. Seeds elevated by turbulence are subjected to the slipstream of the vehicle which may greatly extend dispersal distances compared to tumble dispersal. In addition, this process could interact with dispersal over sealed surfaces of transport corridors by the ambient wind [39].

### Airflow dispersal of species without adaptation to wind dispersal

Our results expand upon previous experiments on vehicle-aided dispersal of *Brassica* and *Ambrosia*. Both species have seeds without obvious morphological adaptations to enhance wind dispersal and accordingly our study found low dispersal distances. Limited dispersal with a maximum distance of 21.5 m was also found for seeds of *Brassica* that were placed on a rural road [17]. Seed transport of *Ambrosia* by vehicles from dense roadside populations resulted in a low density seed rain within the first 25 m of the seed trap transect [45]. Still both species have spread conspicuously along roads [46], [47] and several studies found a strong association of roadside populations of *Brassica* with the direction of traffic flow, e.g. in the direction of processing plants [37], [47], [48]. Hence, dispersal mechanisms other than the slipstream of vehicles appear to enhance long-distance dispersal in these species. In previous experiments we found that spillage of *Brassica* seeds during crop transport [38], might explain distributional patterns at the landscape scale. Also, transport of seeds by mowing vehicles has been shown to be an effective means of *Ambrosia* dispersal along road corridors [45].

### Generality of the experimental results

As the secondary dispersal distances observed in this experiment are on a comparable scale to the primary dispersal distances of our species, secondary HMD by vehicles enhances the spread of these species with a special focus along road corridors. The combination of different processes under field conditions could further increase dispersal distances. Seeds transported by attachment to vehicles could – after detachment - be picked up by the airflow of subsequent vehicles. By causing lateral transport towards suitable germination sites at the road verge, the airflow of vehicles could allow such long distance dispersal to end successfully. Furthermore, other processes such as wind related dispersal [25], [39] or lateral dispersal by vehicles in the opposite driving lane (Fig. 5) could bring seeds back on the driving lane and under the influence of vehicle airflow which would allow repeated dispersal events in the same direction. As speed, mass and shape of vehicles influence their airflow [18], we would expect the effect of vehicles travelling on motorways to be much greater than recorded here. However, seed mortality due to crushing by tyres might also be enhanced by higher volumes of traffic. For a vector-specific analysis of dispersal, these losses could be looked at as the cost of this dispersal process [49].

The observations at our study road were limited to a longitudinal distance of 45 m (and no seeds were found in the turning zone beyond 45 m). While the mode of the dispersal kernel and its decline to this distance could be reliably estimated, the scale of the experiment did not allow for the detection of dispersal events that reach beyond this distance. Further distances could be expected for the wind-dispersed species as most replicates in our experiment recorded seeds in the farthest sections.

In our experiment we used only one car and therefore the time between passes was rather long (>1 minute). In reality traffic is often dense, time intervals are shorter and seeds, especially those with low falling velocities, could be kept in the air for a long time by the flow of traffic. Therefore our experiment could underestimate the dispersal distances compared to the same number of passes by consecutive vehicles.

Finally, the weather conditions may affect the relative importance of different dispersal processes. In our experiment, which was always conducted under dry conditions, a large proportion of the seeds of *Brassica* and *Ambrosia* remained in the lane even after 80 passes. It remains unclear whether such low lateral transport under wet conditions may offer an advantage for long-distance dispersal via attachment to car's surface.

### Conclusions

Human-mediated dispersal has often been characterized as a highly stochastic process that is difficult to quantify, but that has a large effect on long-distance dispersal. Our study contributes to a better understanding of one HMD process, both quantitatively and mechanistically.

Dispersal processes along roads, both anthropogenic and natural, are manifold and include long and short term attachment to car (body versus tyres), vehicle airflow along roads as well as lateral transport, wind dispersal (airborne and sealed surfaces) and tumble dispersal. All these dispersal mechanisms may result in complex interactions considerably enhancing dispersal distances. A better understanding of these interacting dispersal processes may help to increase the accuracy of models of range expansion of invasive species. The high volume of traffic (more than 25 million cars on British roads by 1997 [10]) illustrates the quantitative relevance and potential impact of this dispersal vector within urban areas and beyond. Therefore, HMD in general [1], [3], [14], [15] and vehicle airflow in particular play an important role in understanding and managing invasive species in particular and in conservation ecology in more general. For example, our results on the strong lateral movement of seeds in the slipstream of passing cars might stimulate a changed design of road edges that then hampers lateral movement of seeds from road surface to verge.

## Supporting Information

Video S1 **Video of the study car moving along the study road.** The car was filmed after new seeds of all species were laid out in front of the zero line. The markings indicating the sections parallel and perpendicular to the direction of traffic (Fig. 1) are visible.
(AVI)

Table S1 **Parameter estimates and AIC of the Lognormal and Wald function for dispersal at three different numbers of vehicles passes.**
(DOC)
