# Correction: Human-Mediated Dispersal of Seeds by the Airflow of Vehicles

**DOI:** 10.1371/annotation/50f98e02-214c-4314-b053-ca272101ead1

**Published:** 2013-08-07

**Authors:** Moritz von der Lippe, James M. Bullock, Ingo Kowarik, Tatjana Knopp, Matthias Wichmann

The name of the fifth author was given incorrectly. The correct name is: Matthias C. Wichmann. 

The correct citation is: von der Lippe M, Bullock JM, Kowarik I, Knopp T, Wichmann MC (2013) Human-Mediated Dispersal of Seeds by the Airflow of Vehicles. PLoS ONE 8(1): e52733. doi:10.1371/journal.pone.0052733. 

The correct abbreviation in the Contributions section is: MCW. 

