# Supplementary material for: Human-Mediated Dispersal of Seeds by the Airflow of Vehicles
Source: PLoS One. 2013 Jan 8;8(1):e52733. doi: 10.1371/journal.pone.0052733 (PMC3540062; doi:10.1371/journal.pone.0052733)
Supplement: Table S1 — Parameter estimates and AIC of the Lognormal and Wald function for dispersal at three different numbers of vehicles passes. (DOC) [file pone.0052733.s002.doc]

**Table S1:**
**Parameter estimates and AIC of the Lognormal and Wald function for dispersal at three different numbers of vehicles passes.**

|  |  | ***Lognormal*** | | | ***Wald*** | | |
| --- | --- | --- | --- | --- | --- | --- | --- |
| **Species** | **# of vehicle passes** | *a* (Logn.) | *b* (Logn.) | AIC | *a* (Wald) | *b* (Wald) | AIC |
| *Ailanthus altissima* |  |  |  |  |  |  |  |
|  | 1 | 0.684 | 1.790 | **59.8** | 12.160 | 7.086 | 60.3 |
|  | 20 | 0.730 | 2.146 | 46.5 | 16.744 | 11.817 | **46.3** |
|  | 80 | 0.724 | 2.307 | 43.2 | 20.456 | 12.952 | **43.1** |
| *Clematis vitalba* |  |  |  |  |  |  |  |
|  | 1 | 0.681 | 1.752 | **59.9** | 13.202 | 6.406 | 60.2 |
|  | 20 | 0.700 | 2.151 | 50.2 | 16.846 | 10.838 | **50.1** |
|  | 80 | 0.724 | 2.229 | 45.5 | 18.366 | 12.275 | **45.4** |
| *Ambrosia artemisiifolia* |  |  |  |  |  |  |  |
|  | 1 | 0.616 | -0.476 | **31.6** | 1.371 | 1.000 | 34.1 |
|  | 20 | 0.753 | 0.066 | **39.0** | 1.533 | 1.298 | 39.0 |
|  | 80 | 0.791 | 0.211 | **39.6** | 1.270 | 1.357 | 39.7 |
| *Brassica napus* |  |  |  |  |  |  |  |
|  | 1 | 0.377 | -1.220 | **22.8** | not conv. | not conv. | not conv. |
|  | 20 | 0.641 | -0.435 | **33.0** | 1.358 | 0.995 | 34.6 |
|  | 80 | 0.697 | -0.260 | **34.7** | 1.621 | 1.089 | 35.2 |
